# Association between complement component 4A expression, cognitive performance and brain imaging measures in UK Biobank

**DOI:** 10.1017/S0033291721000179

**Published:** 2022-11

**Authors:** Kevin S. O'Connell, Ida E. Sønderby, Oleksandr Frei, Dennis van der Meer, Lavinia Athanasiu, Olav B. Smeland, Dag Alnæs, Tobias Kaufmann, Lars T. Westlye, Vidar M. Steen, Ole A. Andreassen, Timothy Hughes, Srdjan Djurovic

**Affiliations:** 1 NORMENT, Institute of Clinical Medicine, University of Oslo, & Division of Mental Health and Addiction, Oslo University Hospital, Oslo, Norway; 2 Department of Medical Genetics, Oslo University Hospital, Oslo, Norway; 3 School of Mental Health and Neuroscience, Faculty of Health, Medicine and Life Sciences, Maastricht University, Maastricht, The Netherlands; 4 Department of Psychology, University of Oslo, Oslo, Norway; 5 NORMENT, Department of Clinical Science, University of Bergen, Bergen, Norway; 6 Department of Medical Genetics, Dr Einar Martens' Research Group for Biological Psychiatry, Haukeland University Hospital, Bergen, Norway; 7 Division of Mental Health and Addiction, Oslo University Hospital, Oslo, Norway

**Keywords:** Cognition, immune system, major histocompatibility complex, mental health, psychiatric disorder, schizophrenia

## Abstract

Abstract

**Background:**

Altered expression of the complement component *C4A* gene is a known risk factor for schizophrenia. Further, predicted brain *C4A* expression has also been associated with memory function highlighting that altered C4A expression in the brain may be relevant for cognitive and behavioral traits.

**Methods:**

We obtained genetic information and performance measures on seven cognitive tasks for up to 329 773 individuals from the UK Biobank, as well as brain imaging data for a subset of 33 003 participants. Direct genotypes for variants (*n* = 3213) within the major histocompatibility complex region were used to impute C4 structural variation, from which predicted expression of the *C4A* and *C4B* genes in human brain tissue were predicted. We investigated if predicted brain *C4A* or *C4B* expression were associated with cognitive performance and brain imaging measures using linear regression analyses.

**Results:**

We identified significant negative associations between predicted *C4A* expression and performance on select cognitive tests, and significant associations with MRI-based cortical thickness and surface area in select regions. Finally, we observed significant inconsistent partial mediation of the effects of predicted *C4A* expression on cognitive performance, by specific brain structure measures.

**Conclusions:**

These results demonstrate that the *C4* risk locus is associated with the central endophenotypes of cognitive performance and brain morphology, even when considered independently of other genetic risk factors and in individuals without mental or neurological disorders.

## Introduction

The major histocompatibility complex (MHC) is located on chromosome 6 and is implicated in a number of autoimmune diseases (Howson, Walker, Clayton, & Todd, 2009; Kamitaki et al., 2020; Raychaudhuri et al., 2012). In addition, genetic variants within this region are consistently associated with risk of schizophrenia (International Schizophrenia Consortium et al., 2009; Pardiñas et al., 2018; Schizophrenia Psychiatric Genome-Wide Association Study (GWAS) Consortium, 2011; Schizophrenia Working Group of the Psychiatric Genomics Consortium, 2014; Shi et al., 2009; Stefansson et al., 2009). These associations corroborate serological studies which identified altered levels of inflammatory markers in schizophrenia patients, including complement proteins (Hakobyan, Boyajyan, & Sim, 2005; Laskaris et al., 2019; Maes et al., 1997; Mayilyan, Arnold, Presanis, Soghoyan, & Sim, 2006; Mayilyan, Dodds, Boyajyan, Soghoyan, & Sim, 2008a; Mayilyan, Weinberger, & Sim, 2008b). These findings suggest the involvement of an immune component in psychiatric disorders such as schizophrenia.

In order to better understand the mechanisms underlying the MHC genetic association with schizophrenia, a fine-mapping molecular investigation of the region was conducted and identified that variants within the complement component 4 (*C4*) gene locus are responsible for at least part of the association signal (Sekar et al., 2016). The C4 protein is one of a number of proteins that make up the complement system (Charles, Janeway, Travers, Walport, & Shlomchik, 2001), part of the innate immune system. Complement components were initially shown to modulate neurogenesis in murine primary cortical cell cultures (van Beek et al., 2001). Further investigation of the role of complement components in the central nervous system of genetically modified mice identified its major role in modulating synaptic plasticity (Hong et al., 2016; Stephan, Barres, & Stevens, 2012; Stokowska et al., 2017; Vasek et al., 2016). More recently, complement components were implicated in neuronal migration (Gorelik et al., 2017) and apoptosis (Niculescu et al., 2004) in the central nervous system. Additional evidence for the activity of the complement system in the brain, and its involvement in the pathogenesis of schizophrenia is summarized in recent reviews (Druart & Le Magueresse, 2019; Nimgaonkar, Prasad, Chowdari, Severance, & Yolken, 2017; Tenner, Stevens, & Woodruff, 2018; Woo, Pouget, Zai, & Kennedy, 2019).

The *C4* gene is present as one of two isotypes (*C4A* and *C4B*) and the structural variation between these isotypes, as well as their copy number, was shown to significantly alter the expression level of C4 in post-mortem brain tissue (Sekar et al., 2016). A model of this relationship can be used to predict *C4A* gene expression in the brain based on an individual's genotype. Using this procedure, predicted *C4A* gene expression was associated with risk of schizophrenia in an independent sample (Sekar et al., 2016). Finally, C4 proteins localized to the synapses in post-mortem human brains, and C4 was also demonstrated to modulate synaptic pruning in mice (Sekar et al., 2016), and human-derived neural cultures (Sellgren et al., 2017, 2019).

Independent of these findings, variants within the MHC region were also associated with cognitive performance (Athanasiu et al., 2017; Donohoe et al., 2013; Zhang, Lv, Fan, Tang, & Yi, 2017) and brain structure (Walters et al., 2013) in patients with schizophrenia. Based on these studies, Donohoe et al. (2018) showed that increased predicted *C4A* expression was associated with poorer performance in memory recall measures in a cohort of psychosis patients and healthy controls, as well as in patients only. The direction of effect in control participants was similar to that observed in patients, however, the effect size was smaller and non-significant. In addition, they demonstrated that higher predicted *C4A* expression was associated with lower cortical activity in the middle temporal cortex during visual processing in healthy participants (Donohoe et al., 2018). In support of these findings, complement-dependent synapse elimination was recently identified as a mechanism for memory loss (Wang et al., 2020). These results highlight that *C4A* expression in the brain may be associated with cognitive and behavioral traits not only in patients with psychiatric disorders but also in healthy individuals.

Based on this, our primary aim was to investigate if predicted brain *C4A* expression is associated with cognitive performance in a large adult population-based sample (UK Biobank), without mental or neurological disorders. We hypothesized that higher predicted *C4A* expression would be associated with lower cognitive performance, however, we did not start with any *a priori* assumptions regarding the specific cognitive tasks investigated. Our secondary aims were to investigate if predicted brain *C4A* expression is associated with differences in brain structure and if observed effects on cognitive performance may be mediated by C4A-associated differences in brain structure.

## Methods

### The UK Biobank cohort

The UK Biobank cohort and available data are described elsewhere (Bycroft et al., 2018). Briefly, the UK Biobank project is a prospective cohort study with genetic and phenotypic data collected on approximately 500 000 individuals from across the UK. Multimodal imaging assessments are underway, with magnetic resonance imaging (MRI) of the brain currently available for a subset of individuals (Miller et al., 2016). All data used in this study were obtained from the UK Biobank (http://www.ukbiobank.ac.uk) through application 27412.

We limited the cohort to 409 629 Caucasian individuals (Datafield-22006). This subset is defined as those individuals who self-identified as ‘White British’ and that had similar genetic ancestry based on a principal component analysis (online Supplementary Fig. S1). Individuals with a diagnosed mental or neurological disorder were excluded (Datafields-41202,41204; F/G codes). One from each pair of individuals with a kinship coefficient above 0.053 was also removed prior to analyses (Datafield-2201122012).

The final cohort sample size, after exclusions, with available genetic data was 329 773 (median age 59, range: 40–74). The sample included 152 966 men (median age 59, range: 40–74) and 176 807 women (median age 58, range: 40–71).

All participants provided informed consent prior to enrolment. The authors assert that all procedures contributing to this work comply with the ethical standards of the relevant national and institutional committees on human experimentation and with the Helsinki Declaration of 1975, as revised in 2008.

### Genotyping and quality control

Genotyping of the UK Biobank cohort was performed on two similar arrays. Approximately 50 000 samples were genotyped on the UK BiLEVE array and the remaining 450 000 samples were genotyped on the UK Biobank Axiom array. Further details regarding genotyping and quality control procedures for the UK Biobank are well documented (Bycroft et al., 2018).

### Imputation of C4 structural variation and genetically predicted C4a expression

**Fig. 1.**
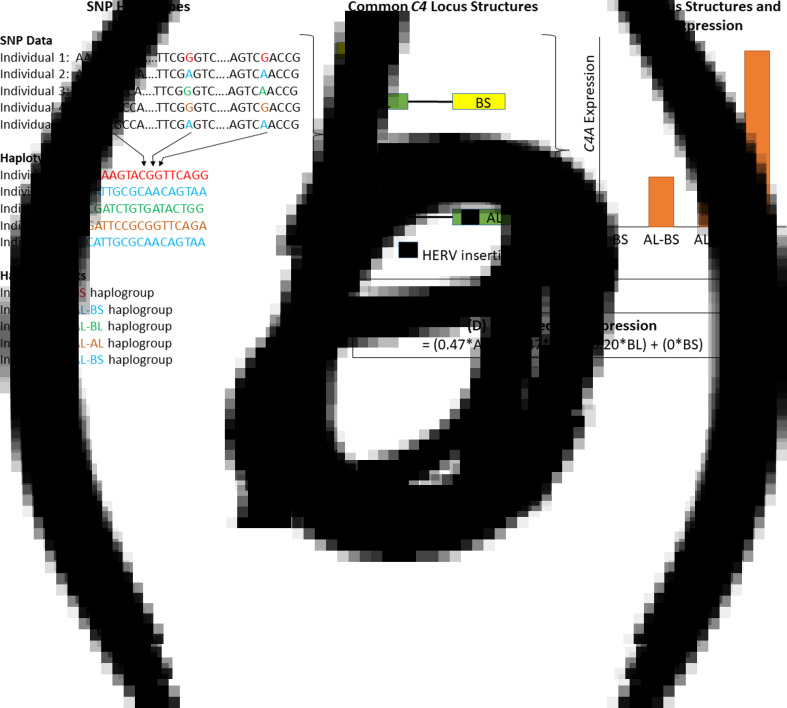
A schematic diagram of the methodology used to obtain predicted expression values for the *C4A* gene within brain tissue, as described by Sekar et al. (2016). First, (*a*) individual genotypes are determined and SNP haplotypes are then inferred from this data. (*b*) The SNP haplotypes can be grouped into haplogroups and each haplogroup corresponds to a specific *C4* locus structure. Four of these structures are common (represented here) and 11 are less common (<10% frequency combined). HERV, human endogenous retroviral insertion. (*c*) Structures with higher copy numbers of *C4A* and *C4L* (both *C4AL* and *C4BL*) isotypes show higher *C4A* expression in brain tissue. (*d*) *C4A* gene expression can be predicted based on the data outlined in panels A–C. AL, AS, BL, and BS refer to the copy number of each of these isotypes in the *C4* locus structure. Structures containing the AS combination are omitted from panels A to C since they are rare, with a frequency of approximately 1% (online Supplementary Table S1) (Sekar et al., 2016). This figure is a schematic and was not generated from actual genotype, expression or other data.

Direct genotypes for variants (*n* = 3213) within the MHC region were used to impute C4 structural variation. This analysis was performed using the 222 haplotype-integrated variant and C4 reference panel (Sekar et al., 2016). The distribution of C4 structural variants was similar to previously described (online Supplementary Table S1) (Sekar et al., 2016; Kamitaki et al., 2020). The imputed C4 structural alleles were then used to determine C4 isotype (C4A, C4B, C4L, and C4S) copy numbers. Here C4A and C4B refer to the two isotypes of the *C4* gene, while C4L and C4S refer to ‘long’ and ‘short’ forms of the gene due to the presence or absence of a human endogenous retroviral (HERV) insertion, respectively. We calculated values for the predicted expression of the *C4A* gene in human brain tissue, based on the previously identified relationship between C4 isotype copy number and *C4A* gene expression (Sekar et al., 2016). The predicted *C4A* expression values ranged between 0 and 2.35 (mean = 1.08, standard deviation = 0.36) (online Supplementary Fig. S2). A summary of this methodology is presented in Fig. 1. Predicted *C4B* expression values were calculated following a similar approach. Predicted *C4A* and *C4B* expression values were used for association with cognitive tasks and brain imaging measures since these variables allow for use of standard linear regression analyses instead of ordinal regression using structural variants.

### Cognitive tasks

We obtained performance measures on seven cognitive tasks from the UK Biobank, and processed them as previously described (Kendall et al., 2017, 2019). Briefly, measures for analysis included the Pairs Matching task (episodic memory, Datafield-399, outcome: total number of errors), the Reaction Time task (simple processing speed, Datafield-20023, outcome: mean reaction time to correct responses), the Fluid Intelligence test (reasoning and problem solving, Datafield-20016, total number of correct answers), the Digit Span task (numeric working memory, Datafield-4282, outcome: maximum number of digits remembered), the Symbol Digit Substitution task (complex processing speed, Datafield-20195, outcome: number of correct substitutions), and the Trail Making A and B tasks (visual attention, Datafields-20156,20157, outcome: time taken to complete these tests). All data were recoded so that higher scores indicate better performance. The number of participants that completed each of these performance measures, with available predicted *C4A* and *C4B exp*ression values and brain imaging data, is provided in Table 1.

**Table 1.** Numbers of participants that completed each of the seven cognitive tasks, with available predicted *C4A* and *C4B* expression values and brain imaging data

Cognitive task	With predicted *C4A* and *C4B* expression (*n*)	With *C4A* and *C4B* expression Values and Brain Imaging Data (*n*)
Pairs matching	329 465	21 989
Reaction time	327 815	22 064
Fluid Intelligence	106 633	7484
Digit span	34 171	2195
Symbol Digit Substitution	81 444	11 696
Trail Making A	71 933	10 427
Trail Making B	71 931	10 427

### Image acquisition and processing

Imaging assessments were conducted at three centers, using the same hardware, software and protocols. A detailed description of the processes for data acquisition, processing and quality control is available (Alfaro-Almagro et al., 2018). The data release from UK Biobank used in this study included 33 003 participants. *C4A* and *C4B exp*ression values were predicted for 27 087 of these participants.

We processed T1-weighted MRI scans from all individuals using the standardized recon-all pipeline of FreeSurfer (Fischl et al., 2002; Fischl, 2012). Furthermore, for each scanner site, we regressed age and sex from the Euler number of both left and right hemispheres and individuals whose Euler numbers were less than 3 standard deviations below the residualized Euler numbers were excluded as outliers (*n* = 618) (Kaufmann et al., 2019). Analyzed brain imaging measures included surface area and mean thickness of 34 cortical regions, total cortical surface area, and mean cortical thickness, the volumes of seven sub-cortical regions, and total intracranial volume (ICV). The total surface area, thickness or volume of each region was calculated by summing the right and left hemispheres.

### Statistical analyses

To determine the relationship between cognitive performance and predicted *C4A* and *C4B* expression, we performed linear regression analyses with each cognitive task as the outcome variable, predicted *C4A* or *C4B* expression as the predictor variable and common covariates, which included age, age-squared, sex, genotyping batch, the first 10 genetic principal components and educational attainment. A summary of the effects of these covariates on *C4A* and *C4B* expression is provided in online Supplementary Table S2. Age-squared was included since this allows the model to accommodate a non-linear relationship between age and the outcome variable if one exists. Educational attainment was determined by the highest qualification obtained by each individual at the time of assessment (Datafield-6138). No significant associations were identified between predicted *C4B* expression and cognitive tasks (online Supplementary Table S3), and therefore predicted *C4B* expression was not tested for associations with brain imaging measures.

To investigate the relationship between brain imaging measures and predicted *C4A* expression values, brain imaging measures were first normalized in R 3.5.0 by an inverse normal transformation of the residual of linear regression on the phenotype correcting for covariates, as previously described (Sønderby et al., 2018). This transformation results in normally distributed covariate-corrected values that were used for downstream analysis. Covariates included the common covariates mentioned above as well as Euler number (Rosen et al., 2018). Regional measures of surface area and mean thickness were corrected for total cortical surface area and total mean cortical thickness, respectively. Subcortical volumes were corrected for ICV.

To determine the association between of predicted *C4A* expression and brain structure, we performed linear regression analyses with the covariate-corrected brain imaging measure as the outcome and predicted *C4A exp*ression as the predictor variable in the model.

**Fig. 2.**
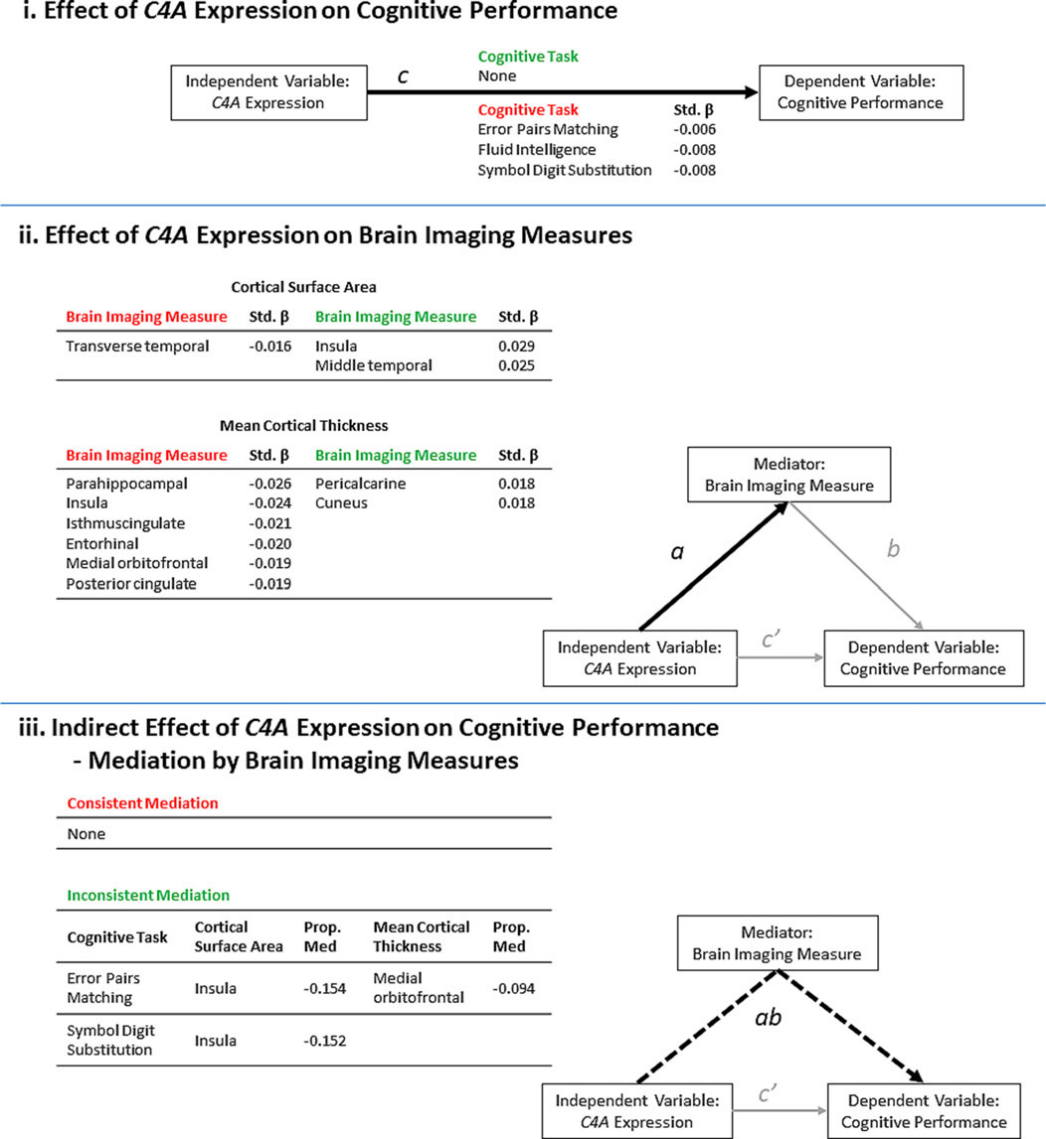
A summary of the results from the significant (FDR <0.05) linear regression models of predicted *C4A* expression values on cognitive performance and brain imaging measures. The results are presented in the context of a mediation model. (i) Higher predicted *C4A* expression was significantly associated with the results from three cognitive tasks. Path *c* = Cognitive task ~ *C4A* expression (ii) Predicted *C4A exp*ression was significantly associated with some measures of cortical surface area and cortical thickness. Path *a* *=* Brain imaging measure ~ *C4A* expression. (iii) A summary of the brain imaging measures identified to significantly mediate the effect of predicted *C4A exp*ression on cognitive performance. Path *ab* = Cognitive task ~ *C4A* expression mediated by brain imaging measures. The proportion of the total effect (Panel i, Path *c*) mediated by changes in the corresponding brain imaging measure is shown (Prop. Med = *ab*/*c*). Negative proportion values indicate inconsistent mediation. Inconsistent mediation occurs when the direction of effect of the direct effect (*c’*) and the indirect effect (*ab*) is in the opposite direction. The standardized *β* (Std. *β*) is shown to indicate the size and direction of effect of higher *C4A* expression on each outcome measure. The green and red headers indicate an increase or decrease in each outcome measure, respectively.

Finally, to determine if the effects of predicted *C4A exp*ression on cognitive tasks were mediated by brain imaging measures, additional linear regression analyses were performed with each cognitive task as the outcome variable, predicted *C4A exp*ression, a regional non-covariate-corrected brain imaging measure and covariates. Covariates included the common covariates, Euler number (Rosen et al., 2018), and educational attainment. Regional measures were corrected for using global measures as described above. Mediation analysis was then performed using the R package mediation v4.4.6, using the bootstrapping method and 5000 simulations per test (Writing Committee for the ENIGMA-CNV Working Group et al., 2019). All significant results are also shown in the context of a mediation model (Fig. 2). A previous study investigating the effects of brain imaging measures on cognitive performance in the UK Biobank has shown significant positive correlations between all of the brain imaging measures included in this study and increased cognitive performance (Cox, Ritchie, Fawns-Ritchie, Tucker-Drob, & Deary, 2019). Those results correspond to path *b* in the mediation analyses performed in this study (Fig. 2).

Since sex-specific *C4A* risk effects were recently identified (Kamitaki et al., 2020), additional analysis was performed as above with the inclusion of an interaction term between *C4A* expression and sex (online Supplementary Table S4). The number of male and female participants that completed each of the performance measures, with available predicted *C4A* and *C4B exp*ression values and brain imaging data, is provided in online Supplementary Table S5.

The distributions of residuals from all models were examined and determined to be normal indicating that linearity assumptions were not violated. Effect sizes reported are the standardized estimates of beta (*β*) from the linear regressions. The partial correlation coefficient (*r*) was computed from the *t*-statistics for the main cognitive and brain structure analyses (online Supplementary Tables S6–S9). The distribution of values for significantly associated cognitive performance tests and brain imaging measures were plotted against ‘binned’ predictions of *C4A* expression levels (online Supplementary Figs S3–S5) and analysis of variance tests and post-hoc Tukey tests were used to determine the differences between these ‘bins’ (online Supplementary Tables S10–S12). Empirical *p* values were converted to False Discovery Rate (FDR) q-values using the R package qvalue v2.14.1. FDR was computed independently for the analyses of cognitive tests (*n* = 7), brain morphology (*n* = 79) and mediation (*n* = 33). Results were considered significant if FDR <0.05. Plots were generated using R library ggplot2 v2.2.1 (Wickham, 2009, p. 2) and the R package ggseg v1.5.1.

## Results

### Effect of *C4a* expression on cognitive performance

Predicted *C4A* expression was significantly (FDR < 0.05) associated with three of the seven cognitive tests (Fig. 2i, Table 2, online Supplementary Table S6). Specifically, higher predicted *C4A* expression was associated with reduced cognitive performance in the pairs matching (Std. *β* = −0.006, *t*-value = −3.28, FDR = 0.009), fluid intelligence (Std. *β* = −0.008, *t*-value = −2.86, FDR = 0.032), and symbol digit substitution (Std. *β* = −0.008, *t*-value = −2.75, FDR = 0.043) cognitive tasks. Analysis of the association between predicted *C4A* expression and cognitive performance measures indicates a linear relationship, not a distinct range of expression above or below which the observed changes occur (online Supplementary Table S10). No significant *C4A–*sex interactions were identified for any of the cognitive tests (online Supplementary Table S4).

**Table 2.** A summary of the results from the significant linear regression models of predicted *C4A exp*ression values on cognitive performance

Phenotype and Covariates	*R*	Std. *β*	Std. Error	Uncorrected *p* value	FDR
Pairs matching					
*C4A* expression	−0.006	−0.006	0.003	1.046 × 10^−3^	9.212 × 10^−3^
Age	−0.128	−0.135	0.675	<1 × 10^−300^	<1 × 10^−300^
Sex (Male)	0.016	0.016	0.002	4.204 × 10^−20^	5.028 × 10^−19^
Fluid Intelligence					
*C4A* expression	−0.009	−0.008	0.016	4.174 × 10^−3^	0.032
Age	−0.011	−0.011	0.001	2.415 × 10^−4^	2.319 × 10^−3^
Sex (Male)	0.066	0.059	0.012	1.209 × 10^−101^	2.838 × 10^−100^
Symbol Digit Substitution					
*C4A* expression	−0.010	−0.008	0.040	5.930 × 10^−3^	0.043
Age	−0.451	−0.455	0.002	<1 × 10^−300^	<1 × 10^−300^
Sex (Male)	−0.007	−0.006	0.029	5.477 × 10^−2^	0.248

All models also included age squared, educational attainment, genotyping batch, and the first 10 genetic principal components as covariates (data not shown). *r* = Partial correlation coefficient. Std. *β* = Standardized *β*. Std. Error = Standard Error.

### Effect of *C4a exp*ression on brain imaging measures

**Fig. 3.**
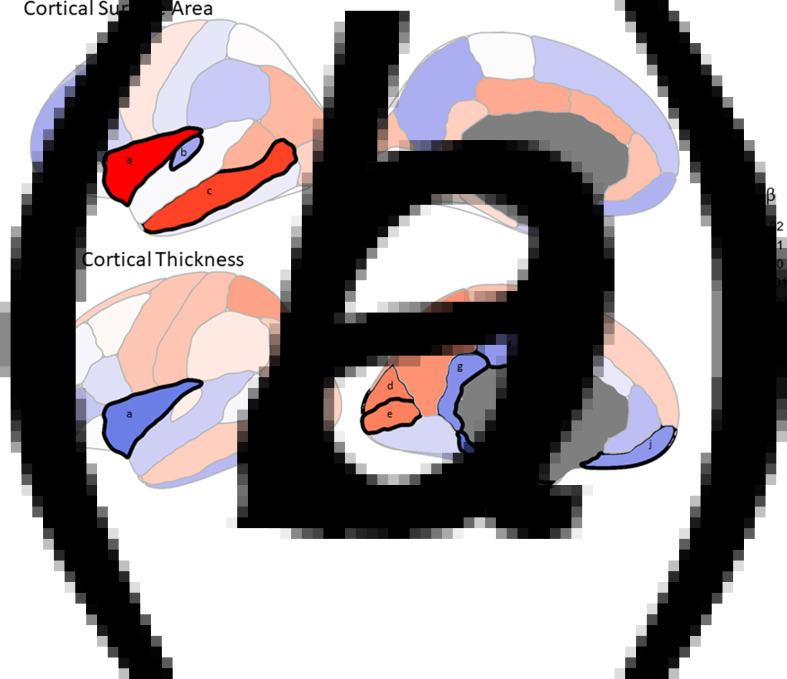
The effect of *C4A exp*ression on regional measures of (*a*) cortical surface area and (*b*) mean cortical thickness. The colors correspond to the standardized *β* (Std. *β*) coefficient for each brain region from the linear regressions. Black demarcations around a brain region indicate that it passes the multiple comparisons–corrected significance threshold of FDR <0.05. a, Insula. b, Transverse temporal. c, Middle temporal. d, Cuneus. e, Pericalcarine. f, Posterior cingulate. g, Isthmuscingulate. h, Parahippocampal. i, Entorhinal. j, Medial orbitofrontal.

Predicted *C4A exp*ression was significantly (FDR < 0.05) associated with three cortical surface area measures (Fig. 3*a*, online Supplementary Table S7). Specifically, higher *C4A* expression was associated with reduced surface area for the transverse temporal measure (Std. *β* = −0.016, *t*-value = −2.68, FDR = 0.045), and increased surface area of the insula (Std. *β* = 0.029, *t*-value = 4.70, FDR = 1.735 × 10^−4^), and middle temporal (Std. *β* = 0.025, *t*-value = 4.15, FDR = 7.458 × 10^−4^) measures, respectively (Fig. 2ii).

When considering mean cortical thickness, predicted *C4A exp*ression was significantly associated with eight measures, the majority (6 of 8) of which were negatively associated with *C4A* expression (Fig. 3*b*, online Supplementary Table S8). Specifically, the parahippocampal (Std. *β* = −0.026, *t*-value = −4.22, FDR = 7.458 × 10^−4^), insula (Std. *β* = −0.024, *t*-value = −3.96, FDR = 1.277 × 10^−3^), isthmuscingulate (Std. *β* = −0.021, *t*-value = −3.38, FDR = 9.865 × 10^−3^), entorhinal (Std. *β* = −0.020, *t*-value = −3.22, FDR = 0.014), medial orbitofrontal (Std. *β* = −0.019, *t*-value = −3.14, FDR = 0.016) and posterior cingulate (Std. *β* = −0.019, *t*-value = −3.08, FDR = 0.017) measures (Fig. 2ii).

No significant associations were identified between predicted *C4A* expression and subcortical volumes. In addition, no other regional brain measures, or global measures including total cortical surface area, total mean cortical thickness and ICV, were significantly associated with predicted *C4A exp*ression (online Supplementary Tables S7–S9). As with cognitive performance, further analysis of the association between predicted *C4A* expression and regional brain imaging measures indicates that this relationship is linear and that there is not a distinct range of expression above or below which the observed changes occur (online Supplementary Tables S11–S12 and online Supplementary Figs S3–S5). Hemisphere-specific results are provided in the supplement (online Supplementary Tables S13–S15). A summary of the effects of predicted *C4A exp*ression on brain imaging measures, and how these results are incorporated into the mediation analyses are shown in Fig. 2ii.

No significant *C4A*–sex interactions were identified for any brain imaging measures (online Supplementary Tables S16–S18).

### Indirect effect of *C4a* expression on cognitive performance – mediation by brain imaging measures

Mediation analyses highlighted that increases in insula surface area and medial orbitofrontal thickness are linked to significant (FDR < 0.05) inconsistent mediation of the effect of higher predicted *C4A exp*ression on two measures of cognitive performance (Fig. 2iii), i.e. the changes in brain imaging measures partially suppress the negative effects of higher *C4A exp*ression on cognitive performance. None of the included brain imaging measures was identified as significant mediators of the effect of predicted *C4A exp*ression on fluid intelligence scores (online Supplementary Table S19).

## Discussion

Here we identified novel significant associations between predicted brain *C4A* expression and cognitive performance in a large adult volunteer sample of individuals without mental or neurological disorders. Additionally, we showed that predicted *C4A* expression was significantly associated with regional cortical thickness and surface area. Further analysis of these associations revealed that their relationships are linear, and that there is no distinct threshold value for predicted *C4A* expression, highlighting that multiple factors likely influence cognition and brain morphology in these individuals within the normal range. Finally, we identified significant inconsistent partial mediation of the effects of *C4A* expression on cognitive performance, by specific brain imaging measures. This indicates that the differences observed in brain morphology may help to protect against *C4A*-associated cognitive deficits. In addition, our observations of lower cognitive performance and differences in brain imaging measures are highly unlikely to be secondary to any mental or neurological disorders or the treatment thereof since we excluded individuals with diagnosed mental or neurological disorders, and the remaining individuals within the UK Biobank tend to be healthier than the general population (Fry et al., 2017).

The main finding of this study is the negative association between predicted *C4A* expression in the brain and episodic memory (Pairs Matching task), reasoning and problem solving (Fluid Intelligence test) and complex processing speed (Symbol Digit Substitution task). Our regression modelling shows that the effects of predicted *C4A* expression, in some instances, are comparable in size to known modifiers of cognitive performance, such as with age for fluid intelligence and with sex for symbol digit substitution (Table 2). As expected, when comparing these effect sizes to those of rare copy number variants (CNVs) with known cognitive effects, a study on the same UK Biobank participants showed that most such CNVs had a greater effect on cognitive performance than that observed for predicted *C4A* expression in this study (Kendall et al., 2019). These results are in line with previous findings, that higher predicted *C4A* expression is associated with poorer performance in memory recall measures in psychosis patients (Donohoe et al., 2018) and that the complement system modulates memory loss (Wang et al., 2020), and further demonstrate that these effects are present within unaffected individuals. Predicted *C4B* expression was not associated with cognitive performance, the effect of the C4 locus was limited to *C4A* as suggested by previous findings (Donohoe et al., 2018; Sekar et al., 2016). Moreover, we did not identify any strong correlation between schizophrenia polygenic risk score and predicted C4A expression (data not shown), implying that predicted C4A expression is not a proxy for schizophrenia polygenic risk in the UK Biobank sample analyzed.

Cognitive impairments reliably distinguish between schizophrenia patients and healthy controls, with large effect sizes in meta-analyses (Mesholam-Gately, Giuliano, Goff, Faraone, & Seidman, 2009). Moreover, similar observations, with smaller effects, for measures of processing speed, attention and memory have also been identified when comparing first-degree relatives of schizophrenia patients to healthy controls (Hou et al., 2016). At a molecular level, shared common variants contributing to both schizophrenia risk and cognitive performance have also been identified (Smeland et al., 2019). These studies highlight cognitive impairment as a core heritable feature of schizophrenia (Barch & Ceaser, 2012; Bora, Yücel, & Pantelis, 2010), which may manifest in both affected patients and healthy individuals with some genetic burden for the disorder. Cognitive deficits have been associated with poorer functional outcomes regardless of age, sex or chronicity of the disorder (Fett et al., 2011). This lead to the suggestion that common mechanisms might modulate individual differences within these cognitive domains, e.g. related to the structure, function and/or connectivity of prefrontal, parietal, cingulate and insula brain regions (Barch & Ceaser, 2012). Our brain imaging results highlight that *C4A* expression may potentially act as one of the causative factors in such mechanisms.

We identified significant associations between predicted *C4A* expression and cortical surface area and/or mean cortical thickness within temporal, cingulate and insula cortex, amongst others (Figs 1ii and 2). In line with previous observations of structural brain abnormalities in patients with schizophrenia (Cobia, Csernansky, & Wang, 2011; van Haren et al., 2011; Kubota et al., 2011; Assunção Leme et al., 2013; Moberget et al., 2018; Alnæs et al., 2019), and more recent associations between schizophrenia polygenic score and structure in unaffected individuals (Alnæs et al., 2019; Neilson et al., 2019; Westlye, Alnæs, van der Meer, Kaufmann, & Andreassen, 2019), higher predicted *C4A* expression was mostly associated with smaller cortical surface area and lower mean cortical thickness (7/11 brain imaging measures, Fig. 2ii). These results, together with our findings on cognitive performance, provide further evidence that some of the common genetic underpinnings of schizophrenia may have similar effects in individuals without mental disorders, in line with dimensional and polygenic risk models (Boyle, Li, & Pritchard, 2017; Purcell et al., 2009; Timpson, Greenwood, Soranzo, Lawson, & Richards, 2018).

In contrast to these results, higher predicted *C4A* expression was also associated with increased cortical surface area and mean cortical thickness in a subset of brain regions (4/11 brain imaging measures, Fig. 2ii). Among these regions with an increased cortical surface area are the insula and the middle temporal cortices. This is contrary to what is observed in schizophrenia patients where the cortical surface area of these regions is reduced (Assunção Leme et al., 2013; Cobia et al., 2011; Kubota et al., 2011; van Haren et al., 2011). Interestingly, however, a larger cortical surface area has previously been identified in unaffected relatives of schizophrenia patients when compared to non-relative controls (Goghari, Rehm, Carter, & MacDonald, 2007). That study showed that relatives had increased gray matter volume and surface area in the left hemisphere, bilaterally in the parahippocampal gyri, and in the left middle temporal lobe, thereby implicating the cingulate and temporal regions which are known to be associated with higher level cognitive, affective, and memory functions (Goghari et al., 2007). The authors suggested two possible explanations for these observed increases in the gray matter of relatives; (i) abnormal cell migration and deficient pruning, and (ii) a protective or compensatory factor against the development of psychosis or loss of associated functioning (Córdova-Palomera et al., 2018; Goghari et al., 2007). Given the molecular functions of complement C4 in the brain, our results could support their suggestion of altered cell migration and synaptic pruning. Moreover, our mediation analyses also suggest the presence of compensatory factors against *C4A*-associated cognitive deficits in individuals without mental disorders.

Previous large scale studies investigating the differences in brain imaging measures between schizophrenia patients and healthy controls show prolific effects of the disorder on numerous measures of cortical surface area and thickness (van Erp et al., 2018), as well as subcortical volumes (van Erp et al., 2016). Although these effects are considered small to medium, they are much larger than the effects of *C4A* expression observed in the present study. Thus, although the changes in brain structure in schizophrenia may be influenced by the level of *C4A* expression, a large number of genetic and environmental factors likely contribute, as suggested by previous studies (Lee et al., 2016).

Brain imaging measures were previously shown to correlate positively with general cognitive performance in the UK Biobank (Cox et al., 2019). Since we had identified a significant negative effect of *C4A* expression on cognitive task performance and significant effects on brain imaging measures (predominantly in the negative direction) (Fig. 2), we expected *ex ante* to observe consistent mediation via the indirect effect (Fig. 2iii, path *ab*), i.e. that some proportion of the effect of *C4A* expression on cognitive performance would be accounted for by the effect of *C4A* expression on brain imaging measures. All of our observations, however, were of inconsistent mediation, i.e. that changes in brain structure, directly or indirectly related to higher *C4A* expression, may act in a protective or compensatory manner against *C4A*-associated cognitive deficits. Significant C4A-associated increases in insula surface area were shown to partially mediate the effects of *C4A* expression on cognitive performance (Fig. 2iii). Specifically, increased insula surface area suppressed the negative effects of *C4A exp*ression on episodic memory (Pairs Matching task) and complex processing speed (Symbol Digit Substitution task) by approximately 15% (Fig. 2iii). Despite the significant correlation identified between *C4A* expression and insula surface area, these mediation results suggest that this relationship is driven by additional components other than *C4A* expression. Rather, the increase in insula surface area is the result of some undetermined mechanism in response to increased *C4A* expression. A similar compensatory relationship was identified between *C4A* expression, cognitive performance, and mean medial orbitofrontal cortical thickness (Fig. 2iii). Increased medial orbitofrontal cortical thickness suppressed the negative effects of *C4A exp*ression on episodic memory (Pairs Matching task) by approximately 9% (Fig. 2iii). In this instance, however, predicted *C4A* expression was negatively associated with mean medial orbitofrontal cortical thickness. Thus, the observed relationship between *C4A* expression and medial orbitofrontal cortical thickness is likely driven by increased *C4A* expression, and the observed protective effect is likely driven by another distinct mechanism in order to compensate for the effects of increased *C4A* expression.

Partial mediation of the effects of *C4A* expression on cognitive performance, by changes in brain imaging measures, suggests that additional mechanisms play a role in modulating this relationship. Furthermore, given the healthier bias of UK Biobank participants (Fry et al., 2017), further exaggerated by our removal of individuals with mental or neurological disorders, it is tempting to speculate that these participants may share other protective or compensatory factors, in addition to the brain imaging differences identified in this study, which might mask the true effect of *C4A* expression on cognitive performance. Thus, the true effect would likely be greater in an unbiased population cohort. Future studies should identify additional factors associated with changes in *C4A* expression and cognitive performance in order to determine other mechanisms that might contribute to their relationship.

A limitation to the current study is that the UK Biobank has an older age distribution in comparison to patients included in most schizophrenia studies, which are commonly conducted on individuals within an age range more closely matching the age of onset of the disorder (18–25 years). As a result, despite controlling for age in our analyses, we cannot exclude a potential effect of aging on the results. Studies in prospective cohorts are required to address this limitation. A second limitation is the reduced sample size for some of the cognitive tasks. Since the identified significant effects of *C4A* expression of cognitive tasks were small, and predominantly identified for those tasks with the largest sample sizes, these reduced numbers may have resulted in false negatives. Future studies with larger samples for these cognitive tasks are required to determine their true relationship with *C4A* expression. Finally, the significant effects of *C4A* expression on cognitive performance and brain morphology identified in this study are very small. By comparison, the effects of brain imaging measures on cognitive performance are magnitudes greater than the effects of *C4A* expression on cognitive performance (online Supplementary Table S20). This highlights that a large number of additional genetic and environmental factors contribute to these phenotypes.

In conclusion, we observed that higher predicted *C4A* expression is associated with lower cognitive performance and regional cortical surface area and thickness. Moreover, we provide evidence that the observed changes in cognitive performance, as a result of predicted *C4A* expression, may be mediated by C4A-associated changes in brain structure. These results demonstrate that *C4* locus affects cognition and brain morphology in individuals without mental or neurological disorders.

## Supplementary material

For supplementary material accompanying this paper visit https://doi.org/10.1017/S0033291721000179. click here to view supplementary material
