# Supplementary material for: Association between complement component 4A expression, cognitive performance and brain imaging measures in UK Biobank
Source: Psychol Med. 2021 Mar 3;52(15):3497–507. doi: 10.1017/S0033291721000179 (PMC9772918; doi:10.1017/S0033291721000179)

Supplementary Figures

Supplementary Figure 1. Principal component scatter plot showing the distribution of individuals included from the full UK Biobank cohort (n = 329,773). The first and second principal components for each individual on the x and y axes, respectively

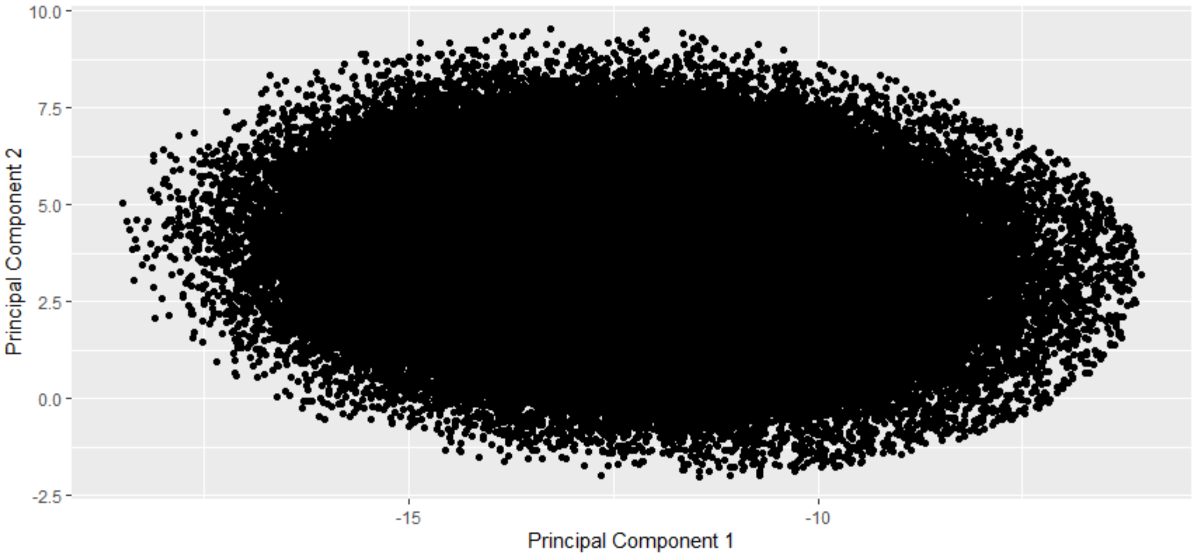

Supplementary Figure 2. Histograms showing the distribution of estimated *C4A* expression values in (A) the full UK Biobank cohort (n = 329,773) and (B) in the subset of the UK Biobank cohort with available imaging data (n = 27,087).

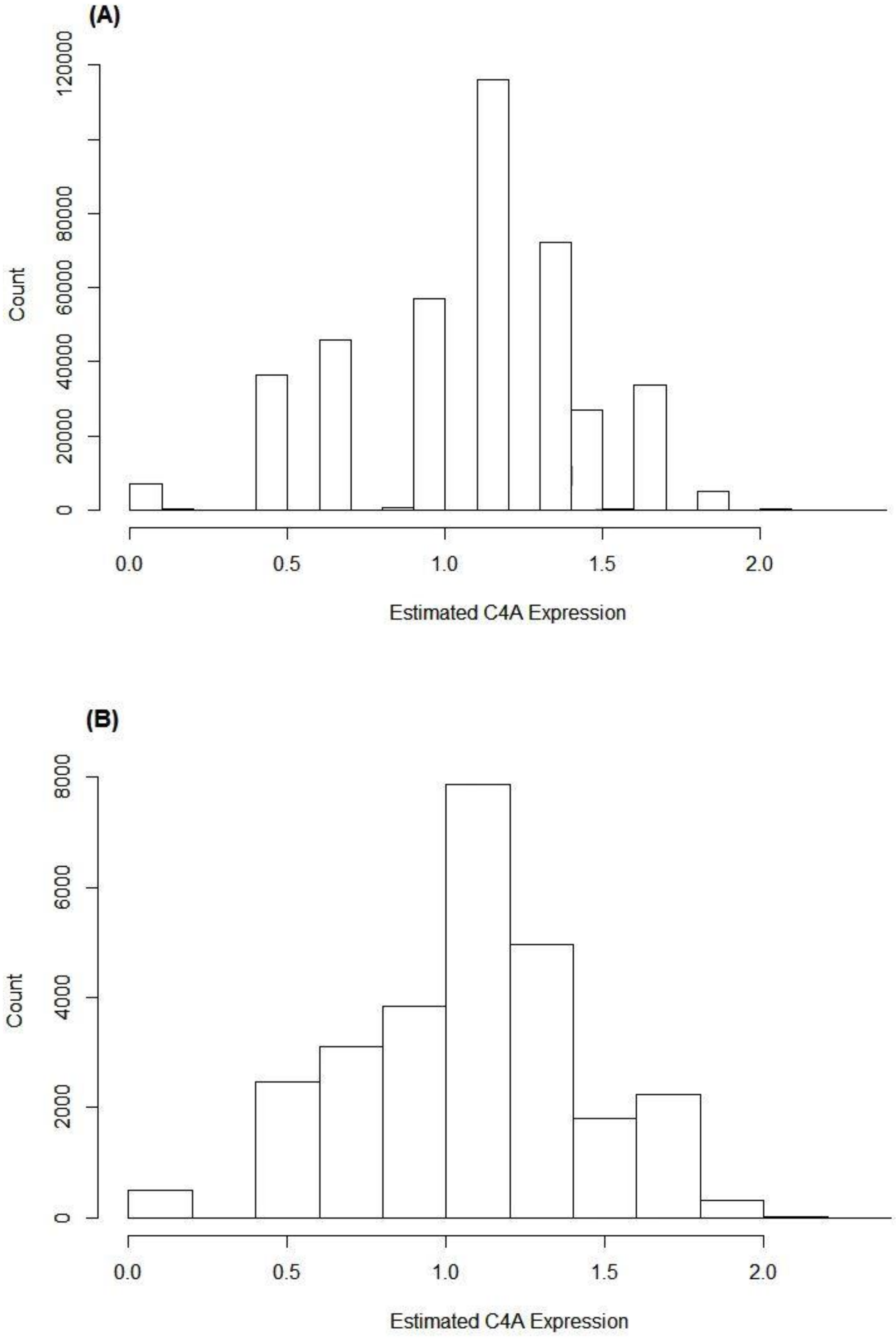

Supplementary Figure 3. Scatter plots for each of the cognitive tasks associated (FDR < 0.05) with estimated *C4A* expression. Box plots for each 'bin' of distinct estimated *C4A* expression values are also shown. Values for the cognitive tasks are plotted on the y-axis and estimated *C4A* expression (C4exp_val) is plotted on the x-axis.

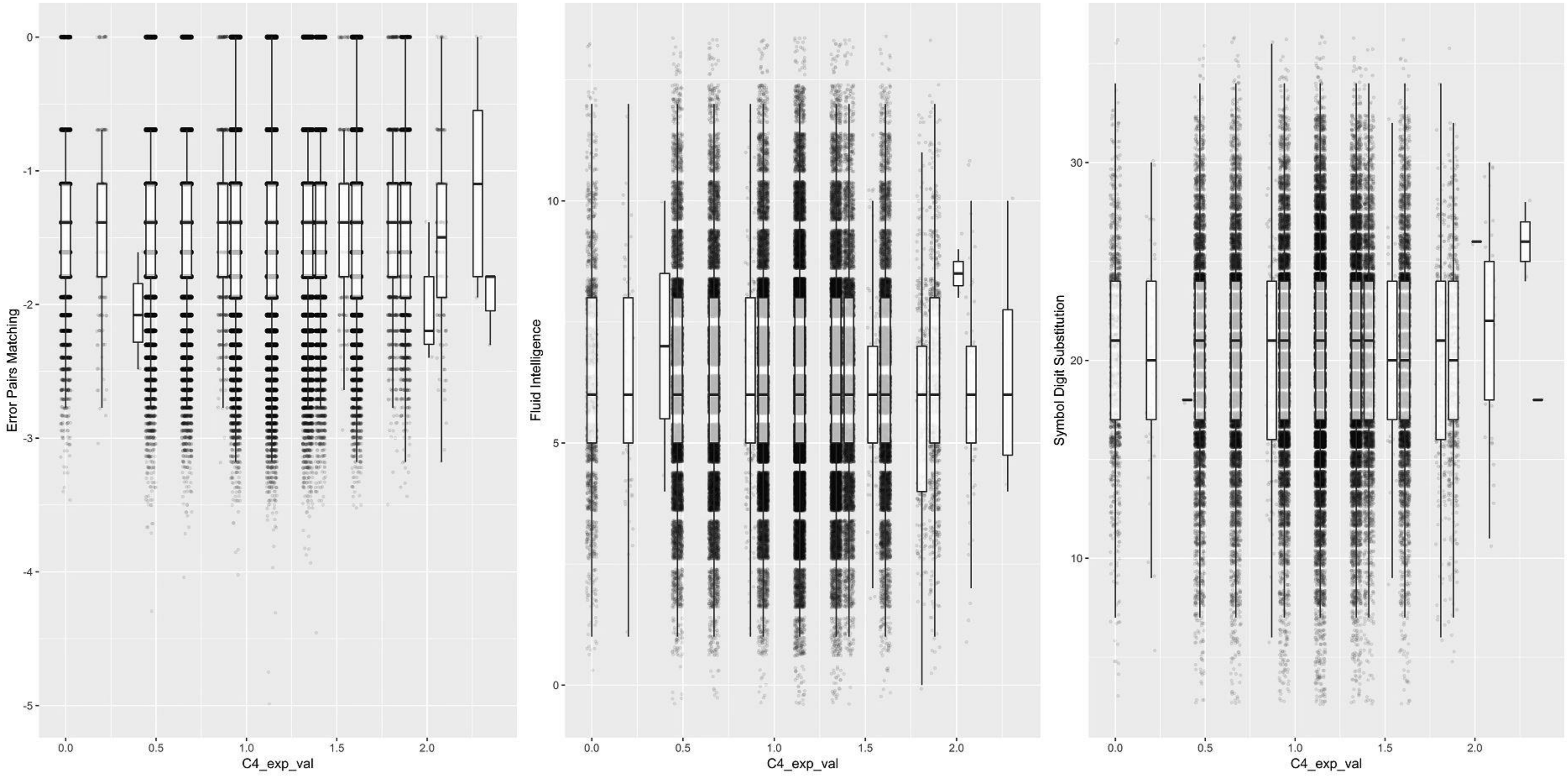

Supplementary Figure 4. Scatter plots for each of the regional cortical surface area measures associated (FDR < 0.05) with estimated *C4A* expression. Box plots for each 'bin' of distinct estimated *C4A* expression values are also shown. Values for cortical surface area are plotted on the y-axis and estimated *C4A* expression (C4exp_val) is plotted on the x-axis.

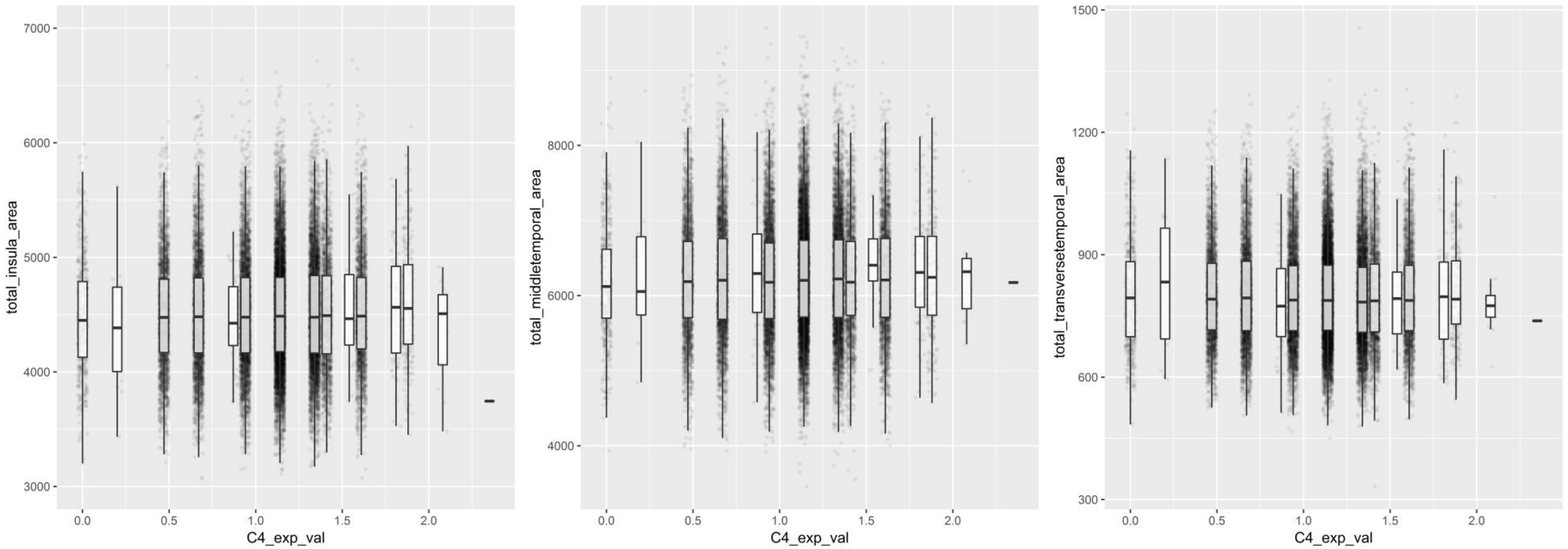

Supplementary Figure 5. Scatter plots for each of the regional cortical thickness measures associated (FDR < 0.05) with estimated *C4A* expression. Box plots for each 'bin' of distinct estimated *C4A* expression values are also shown. Values for mean cortical thickness are plotted on the y-axis and estimated *C4A* expression (C4exp_val) is plotted on the x-axis.

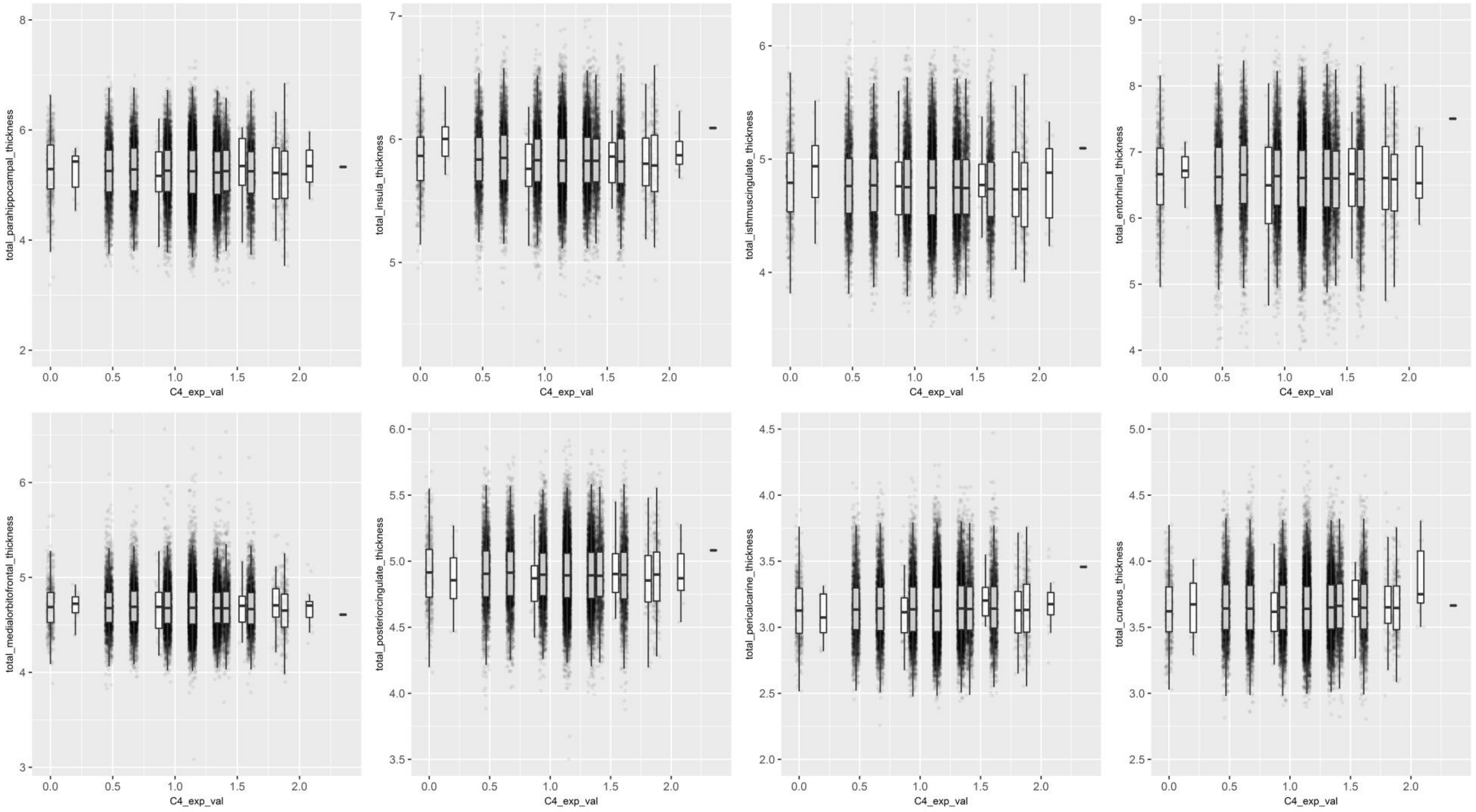

Supplement: Supplementary file 1 [file S0033291721000179sup.zip › S0033291721000179sup001.pdf]
